# Monitoring for neovascular age-related macular degeneration (AMD) reactivation at home: the MONARCH study

**DOI:** 10.1038/s41433-020-0910-4

**Published:** 2020-05-04

**Authors:** Elizabeth Ward, Robin A. Wickens, Abby O’Connell, Lucy A. Culliford, Chris A. Rogers, Eleanor A. Gidman, Tunde Peto, Paul C. Knox, Benjamin J. L. Burton, Andrew J. Lotery, Sobha Sivaprasad, Michael Donnelly, Charlene Treanor, Ruth E. Hogg, Barnaby C. Reeves

**Affiliations:** 1 Bristol Trials Centre (CTEU), University of Bristol, Bristol Royal Infirmary, Bristol, BS2 8HW UK; 2 Centre for Public Health, Queen’s University of Belfast, Belfast, BT12 6BA UK; 3 Department of Eye and Vision Science, University of Liverpool, Liverpool, L7 8TX UK; 4 James Paget University Hospitals NHS Foundation Trust, Norfolk, NR31 6LA UK; 5 Department of Clinical and Experimental Sciences, Faculty of Medicine, University of Southampton, Southampton, SO16 6YD UK; 6 NIHR Moorfields Biomedical Research Centre, Moorfields Eye Hospital NHS Foundation Trust, London, EC1V 2PD UK

**Keywords:** Macular degeneration, Outcomes research, Eye manifestations

## Abstract

**Aims:**

This study aims to quantify the diagnostic test-accuracy of three visual function self-monitoring tests for detection of active disease in patients with neovascular age-related macular degeneration (nAMD) when compared with usual care. An integrated qualitative study will investigate the acceptability of these home-based testing strategies.

**Methods:**

All consenting participants are provided with an equipment pack containing an iPod touch with two vision test applications installed and a paper journal of reading tests. Participants self-monitor their vision at home each week with all three tests for 12–18 months. Usual care continues over this period. Key eligibility criteria are: age ≥50 years; at least one eye with AMD with ≥6–≤42 months since first AMD treatment; and vision not worse than Snellen 6/60, LogMAR 1.04 or 33 letters. The primary outcome, and reference standard, is diagnosis of active disease during usual care monitoring in the Hospital Eye Service. Secondary outcomes include duration of study participation, ability of participants to do the tests, adherence to weekly testing and acceptability of the tests to participants.

**Conclusions:**

Recruitment is in progress at five NHS centres. Challenges in procuring equipment, setting up the devices and transporting devices containing lithium batteries to participating sites delayed the start of recruitment. The study will describe the performance of the tests self-administered at home in detecting active disease compared to usual care monitoring. It will also describe the feasibility of the NHS implementing patient-administered electronic tests or similar applications at home for monitoring health.

## Introduction

Wet age-related macular degeneration (neovascular AMD, nAMD) is the leading cause of vision loss in people aged ≥50 years [1]. Current treatment for nAMD is intravitreal injections of anti-angiogenic drugs that inhibit vascular endothelial growth factor (anti-VEGF antibodies). Treatment starts with a loading phase of 3 injections at intervals of 4–6 weeks; patients then enter a maintenance phase and are reassessed at each subsequent hospital monitoring visit to determine lesion activity. The two main treatment regimens are pro re nata (prn; treatment as required) and treat-and-extend (prophylactic injections and increasing the time interval between visits up to 3 months). The frequency of monitoring depends on the drug prescribed and the treatment regimen. Lesion status can become inactive in a proportion of eyes during the maintenance phase, which are then treated prn or by treat-and-extend. Relapse is common with prn treatment and can still occur with a treat-and-extend regimen.

Monitoring visits use a combination of best-corrected visual acuity (BCVA), clinical biomicroscopic examination and optical coherence tomography (OCT) or other retinal imaging modalities to determine if a lesion is active (wet) or inactive (dry). Patients may have many months without requiring treatment and regular monitoring visits place a significant burden on NHS Hospital Eye Service (HES) out-patient clinics [2]. Self-monitoring at home (“home-monitoring”) for patients with inactive disease would be more convenient and less costly for both patients and the NHS. If self-monitoring were to identify reactivation accurately, HES clinic appointments could be given only given to patients likely to require treatment or to newly referred patients, enabling prompt initiation of treatment.

Home-monitoring with internet-enabled devices is possible due to development of applications for self-monitoring visual function in nAMD [3]. Such applications allow automatic transmission of test results for review without the need for patients to interpret test results [4, 5]. However, reactivation of nAMD is difficult to detect because inactive lesions nevertheless cause some degree of visual disability. Therefore, any test must detect an *increase* or *change* in visual disability, not just the *presence* of disability. Technologies such as visual and memory stimulating grids [6], preferential hyperacuity perimetry [4, 7] and shape discrimination tests [5, 8–11] have been reported to quantify distortion more accurately than either the Amsler grid or visual acuity in clinical settings [3].

The MONARCH study is evaluating three home-monitoring tests. All have some supporting peer-reviewed literature and usability data from AMD populations [5, 6, 8, 9, 12–15]. Two of these tests are electronic applications that run on an iPod touch and one is a paper journal of reading puzzles.

## Objectives

The objectives of the MONARCH study are:

- To estimate the test accuracy of three tests to self-monitor reactivation of nAMD compared to the reference standard of detection during usual care monitoring in HES clinics.
- To determine the acceptability of the tests to patients and carers, explore reasons for non-adherence and the influence of carers on the acceptability of the tests and adherence.
- To explore whether inequalities (by age, sex, social-economic status and visual acuity) exist in recruitment to the study, and are associated with participants’ ability to do the tests and to adhere to weekly testing.
- To provide pilot data for using the same home-monitoring tests to detect conversion to nAMD in fellow eyes of patients with unilateral disease, compared to the reference standard of detecting conversion in usual care monitoring.

## Subjects and methods

### Study design

**Fig. 1:**
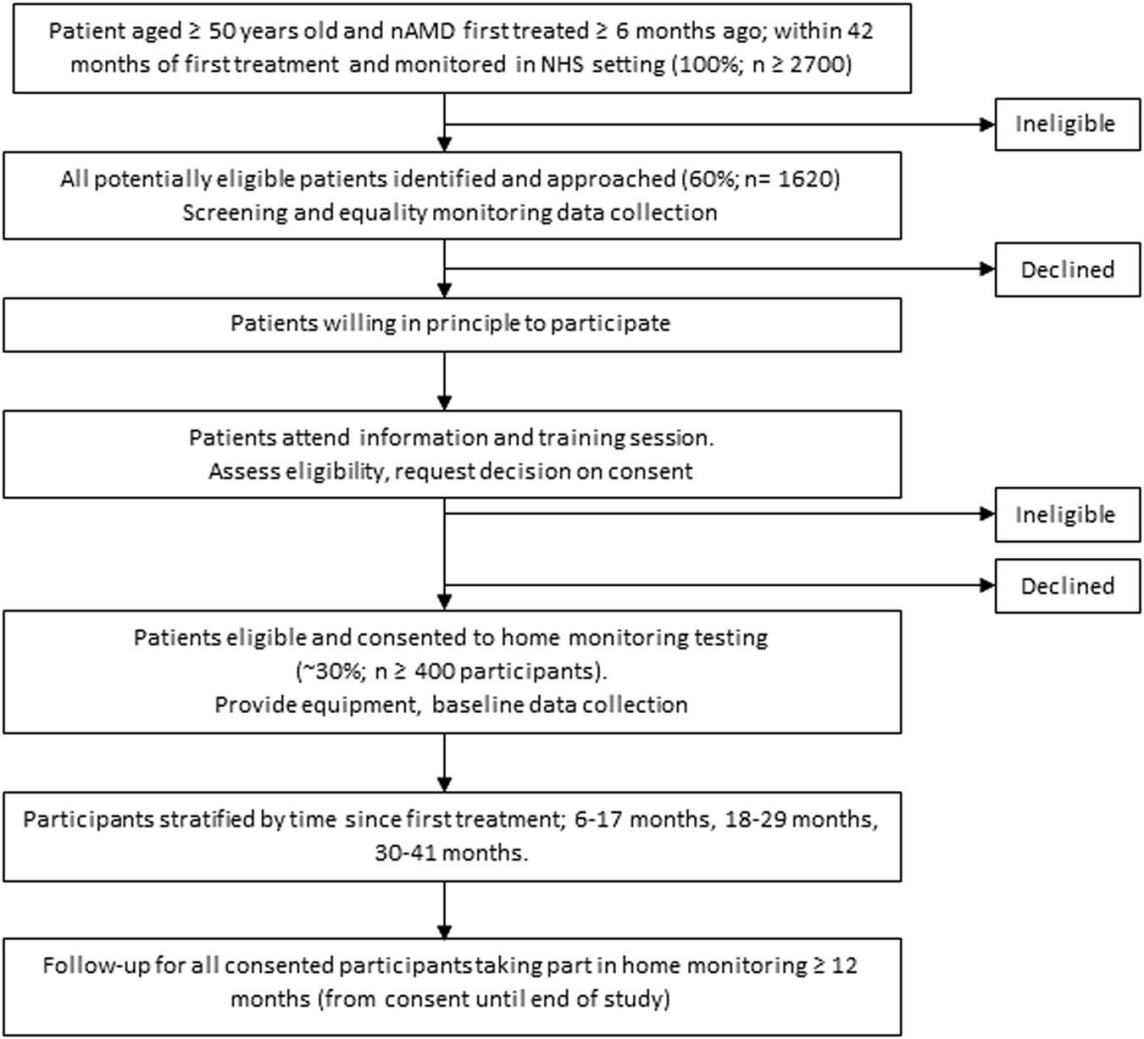
Study schema. The study schema shows the recruitment pathway, predicted numbers and follow-up schedule.

The MONARCH study is a multicentre, diagnostic test-accuracy cohort study. Participants are recruited in secondary care hospital eye service (HES) clinics. Eligible patients are asked to use three home-monitoring vision tests (the index tests) to test their vision weekly at home for a period of 12–30 months. Participants continue to attend HES monitoring clinics with retinal imaging carried out as required to inform usual care management decisions. See Fig. 1 for the study schema and Supplementary Information for details of retinal image collection.

There are no safety reporting procedures for this study; the study is regarded as low risk as it does not require participants to undergo any additional investigations and clinical adverse events cannot be attributed to study specific procedures.

All participating sites are NHS Trusts based in the United Kingdom (UK). The study has a favourable opinion from the Northern Ireland Health and Social Care Research Ethics Committee A (ref 17/NI/0235), is approved by the Health Research Authority, England and is being conducted in accordance with established principles of Good Clinical Practice [16].

### Integrated qualitative study

Three sites are also taking part in an integrated qualitative study (objective B). Person-centred interviews are capturing difficulties, concerns, fears and perceived benefits about the index tests. Information about factors that influence successful implementation for specific participant subgroups is also being collected. A qualitative researcher is conducting (i) one-to-one home-based interviews and observations of participants regularly performing the index tests (*n* = ~75), (ii) telephone or e-interviews with other subgroups (see *Study Population* below), and (iii) interviews with health-care professionals involved in the recruitment and training of participants at each site.

## Study population

Patients with at least one potential study eye are eligible. A study eye must have a diagnosis of nAMD meeting the eligibility criteria. Participants with bilateral nAMD may have two study eyes if both meet the criteria. A fellow eye is an eye without nAMD, but which meets all other eligibility criteria. An excluded eye is an eye which is not eligible to be a study eye or a fellow eye.

Inclusion criteria

A participant aged ≥ 50 years old with at least one potential study eye is eligible if:

1. Eye was first treated for active nAMD ≥6 months ago and ≤42 months ago,
2. Eye is currently being monitored for nAMD by the NHS.

Exclusion criteria

A potential *study eye* is excluded if ANY of the following apply:

1. Vision is worse than Snellen score 6/60, LogMAR 1.04 or 33 letters;
2. Vision is limited by a condition other than nAMD;
3. Surgery in the potential study eye in the previous 6 months;
4. Refractive error in the potential study eye > −6D;
5. Retinal or choroidal neovascularisation in the potential study eye not due to nAMD.
  In addition, a *participant* is excluded if ANY of the following apply:
6. Unable to do one or more of the proposed tests during the training session;
7. Unable to understand English;
8. Home or personal circumstances unsuitable for home testing.

Participants complete the index tests for both study and fellow eyes. Eye-specific outcomes, such as visual acuity, are collected at usual care monitoring visits. An excluded eye can convert to a study eye if time since first treatment and/or time since surgery reaches 6 months and all other eligibility criteria are met.

Participants are provided with all equipment required for the study. Details of all patients approached and reason(s) for non-participation are documented. A minimum dataset comprising age, sex, visual acuity, index of multiple deprivation and ethnicity is collected for all patients approached to monitor inequalities for objective C. Patient approach is detailed in Supplementary Information.

### Study population: integrated qualitative study

Eligibility is not separately assessed for the integrated qualitative study. Participants and carers who consent to be contacted are approached directly by the qualitative research team. Health care professionals willing to be interviewed are invited for face-to-face, telephone or e-interview to explore acceptability of study processes and reasons why patients decline the study. Maximum-variation and purposive sampling will capture the range of participant and related-factors that may be potentially relevant to assessing acceptability (e.g. age, gender and eye health history) and the role of carers. Patients will be asked to ‘nominate’ a carer or person who offers personal support to them. The qualitative sample comprises a number of subgroups, described in Supplementary Information.

## Stratification

Participants are being recruited into 3 strata according to time since first treatment for nAMD in a study eye (first-treated study eye for participants with two study eyes): (a) 6–17 months; (b) 18–29 months; (c) 30–41 months. This will allow the performance of index tests to be evaluated across a range of nAMD durations. Approximately equal recruitment into each stratum is planned.

## Home-monitoring (index) tests

The following tests are being evaluated: (a) KeepSight Journal (KSJ) [6] adapted by the study team for UK use, (b) MyVisionTrack® (mVT) electronic application [5, 8, 9, 12, 15] (Genentech Inc.) and (c) MultiBit (MBT) electronic application [13, 14], developed by Visumetrics, licensed by Novartis International AG.

**Fig. 2:**
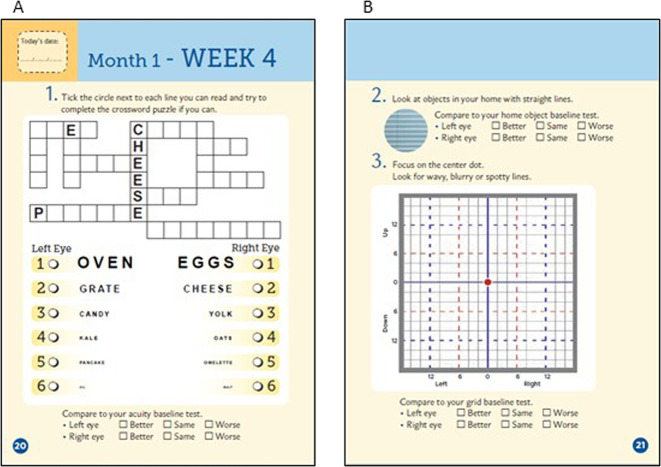
KeepSight journal puzzles for measuring visual acuity. Participants complete the paper KeepSight journal, containing **(a**) a reading vision test, (**b**) a daily home object test, and (**c**) a grid test, weekly.

The paper-based KSJ was developed by the International Macular and Retinal Foundation (New Gloucester, Maine, US). It comprises three tests, viewed one eye at a time: (a) near visual acuity formatted as a puzzle with varying font sizes (Fig. 2a), (b) an instruction to assess distortions by viewing objects with straight lines (Fig. 2b), (c) a modified Amsler chart to record areas of distortion or scotoma (Fig. 2b).

**Fig. 3:**
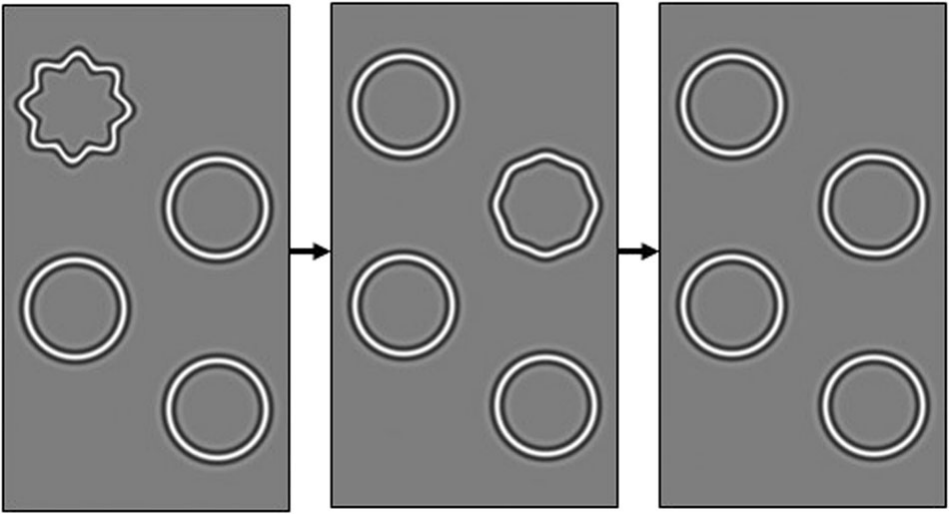
MyVisionTrack® (mVT) electronic software application. Participants select the odd circle out, i.e. irregularly shaped circle; completing multiple rounds which increase in difficulty.

mVT is a shape discrimination threshold test displayed on an iPod Touch. It displays four circles, one of which is deformed. The participant identifies the odd-one-out (Fig. 3).

**Fig. 4:**
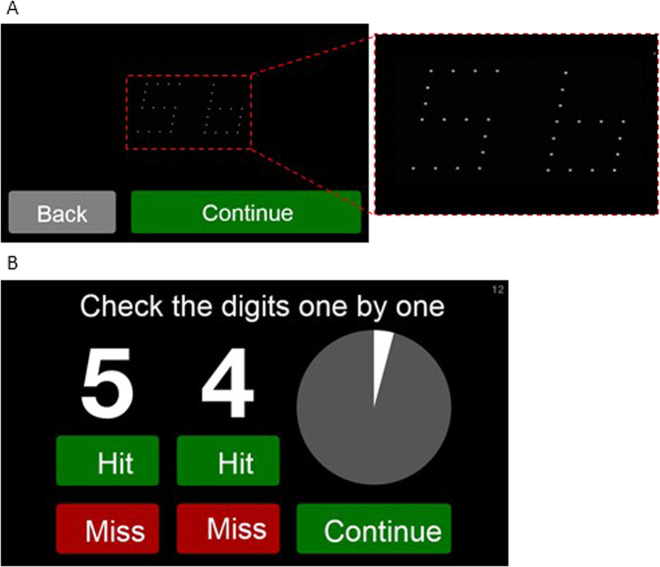
MultiBit (MBT) electronic software application. **a** Pairs of numbers are displayed on the screen in quick succession. Participants must say the numbers they see aloud whilst the iPod audio-records. **b** At the end of the test, the numbers which were presented are displayed clearly and the audio-recording is played back to the participant. Participants have to mark their performance; if they said the correct number, they select the ‘Hit’ button below the corresponding number or if they said an incorrect number, they select the ‘Miss’ button.

MBT is a near acuity threshold test, which displays numbers made up of receptive field size dots or ‘rarebits’. These stimuli provide limited information to the visual system compared to conventional targets. Patients are presented with pairs of numbers (Fig. 4a) and required to state aloud the numbers they can see. The numbers are then presented in high contrast with a recording of the participant’s responses and the participant marks their own performance (Fig. 4b). We are validating self-marking in a sample of patients.

## Reference standard

The reference standard is the status of the lesion in study eyes during usual care monitoring in HES clinics, classified as: definitely active, definitely inactive, or uncertain. Lesion status is decided by the ophthalmologist at the clinic visit and not defined by tests results, although optical coherence tomography (OCT) imaging and visual acuity will typically be available to complement clinical examination. Local site teams collect data for study and fellow eyes at usual care monitoring visits when the status of an nAMD lesion and/or the treatment plan is reviewed, i.e. a “management decision” is made. See Supplementary Information for the data collection schedule. Data are also collected on anti-VEGF injections administered between usual care monitoring visits.

## Participant self-reported data

A participant’s willingness to continue in the study, wish for retraining and self-reported data (e.g. a participant’s own assessment of their vision) are confirmed by phone or in person by the local research team before each management decision in usual care.

## Equipment and technical support

Participants are provided with an equipment pack containing: an eye patch, an iPod with mVT and MBT installed and activated, a mobile broadband device (MiFi device), a lens cloth, a stylus pen, a paper KSJ, a pen, instruction sheets, a weekly checklist and a phone number for technical support.

The Clinical Trials and Evaluation Unit (CTEU) study management team receives calls to the technical support phone line during standard office hours Mon-Fri (except holidays). Calls received to the support line are documented and reasons reviewed.

## Adherence to home eye testing

Adherence is monitored from test data transmitted to the CTEU Bristol. Data summaries are reported to the study management group and study steering committee. Non-adherent participants who have provided consent for telephone contact are called by CTEU Bristol to ‘trouble-shoot’. Reasons for non-adherence are being documented.

## Outcomes

### Primary outcome

The primary outcome is classification of lesion status in a study eye.

### Secondary outcomes

For Objective C we are measuring outcomes of uptake of index tests:

(a) Participation in the study, defined as consent (yes/no) among eligible patients approached to take part.

(b) Ability of participants to do the tests during follow-up, defined as the proportion of HES monitoring visits for which some data for an index test are available since the last HES monitoring visit.

(c) Adherence of participants to weekly testing, defined as the proportion of weeks for which data for an index test are available, aggregated across intervals between monitoring visits.

For Objective D, the outcome is onset of active nAMD in fellow eyes, defined by initiation of treatment.

## Masking

All personnel carrying out usual care NHS monitoring are masked to data from index tests. Site teams are instructed to report any instance when a participant’s index test results are seen by a member of the site team, for example when assisting a patient who is having difficulties with the equipment/tests, as a protocol deviation. Reasons for deviations are recorded and remedial actions implemented. Participants may gain an impression that their vision has changed from home-monitoring tests.

## Features to minimise bias

Risk of bias is considered with respect to bias domains previously described for diagnostic accuracy studies [17]. Features of the study to protect against bias in each domain are described in Supplementary Information.

## Withdrawal and discharge

Each participant can withdraw from the study at any time. An investigator may also withdraw a participant at any time. If a participant is discharged from usual NHS monitoring, the participant is withdrawn from the study. All withdrawals are recorded. Data collected up to withdrawal will be used in analyses, unless consent is withdrawn.

Participants can choose to stop completing one or two of the index tests and continue in the study. The numbers of participants withdrawn, lost to follow-up or deceased will be reported.

## Sample size

### Objectives A and C

The target sample size is 400 participants with matched data from home eye testing. The number of visits is the key parameter and the target is based on assumptions that: participants will have about 2300 monitoring visits (average 6 visits/participant, with 5% attrition); nAMD will be ‘active’ for 30% of monitoring visits; and correlations between tests and reference standard will be 0.6 for both active and inactive lesions [18]. Multiple visits per participant are not independent and measurement error will dilute power to discriminate test performance; therefore, we have assumed an effective sample size of 1200 visits. This number of visits gives 90% power to detect a difference of 0.06, or 80% power to detect a difference of 0.05, in the area under the receiver operating characteristic curves (AUROC) for two tests if the AUROC is 0.75 [18].

### Objective D

Estimates of the rate of conversion to nAMD per year among fellow eyes vary, ranging from 4 to 16% [19–22]. Assuming the risk in unselected patients is 5–6% per year, about 50 patients will have nAMD in both eyes at the time of recruitment. Among the remaining 350 patients, we expect fellow eyes of about 25–30 patients to convert to nAMD.

## Plan for statistical analysis

For the index texts, alternative threshold criteria for classification will be explored to maximise test performance.

### Objective A

The study will be analysed and reported in line with reporting guidelines for studies of diagnostic accuracy [23] and will follow a statistical analysis plan that will be written in advance of the analyses being carried out. We are unable to prespecify the analyses in detail because of the early stage of evaluation of the index tests.

Sensitivity, specificity, positive and negative predictive values of each test will be reported with 95% confidence intervals. The overall performance of the tests will be quantified by the AUROC. AUROCs for the tests will be compared to determine if one or more tests is superior to one or more of the others. Analyses will take account of the structure within the data, i.e. the nesting of visits (and eyes) within patients.

Other analyses may be explored to investigate whether the performance of home-monitoring overall can be improved by, for example, combining information: (a) from multiple index tests; (b) from adjacent home-monitoring periods preceding a monitoring visit (to see if there is evidence that index tests provide ‘advance warning’ of nAMD becoming active); (c) for the study eye and an unaffected fellow eye (to see if differences in scores between affected and unaffected eyes contribute to test accuracy). See Supplementary Information for further details.

### Objective B

Interviews will be audio-recorded and transcribed for analysis and reporting. The research team will review results iteratively and data will be managed and analysed using NVivo software and content and thematic analytical strategies [24, 25]. The focus of the analysis will be on, for example, the acceptability of the tests, the factors that facilitate or impede such acceptability, ease/difficulty of using each test and the perceived benefits as well as the role of carers and family members [26]. The transcripts of interviews will be analysed to produce an integrated and synthesised account and interpretation of the acceptance of the new tests. The qualitative researchers and wider research team will meet to discuss iteratively and early on the results of the analysis including the generation of codes and categories from the content of the transcripts [27]. Overall, the rigour, transparency and sensitivity of the methodology [28] will be enhanced by following the consolidated criteria for reporting qualitative research (COREQ) such as respondent validation, reflexivity and discussion of analytical codes and categories [29].

### Objective C

Regression models will be fitted to explore the influences of age, sex, social-economic status and visual acuity at recruitment on the outcomes of: consent to take part (among all patients approached); ability of a participant to complete a test, analysed separately for each index test (among all participants); and adherence to the study protocol (among all participants). The influence of these factors will be reported as odds ratios with 95% confidence intervals. Analyses will take account of nesting of visits within participants.

### Objective D

All analyses for objective D will be descriptive only. We will explore how test accuracy for detecting conversion changes as a function of index test scores and report the test accuracy statistics for each test for detecting conversion, with 95% confidence intervals.

### Frequency of analyses

For study objectives A, C and D, the primary analysis will take place when follow-up is complete for all recruited participants. No formal interim analysis is planned.

Data for objective B will be analysed iteratively during the study and there are no planned subgroup analyses. Study findings will be reported descriptively by strata.

## Study management and monitoring

See supplementary information for details.

## Protocol amendments

Version 3.0, in use since 30/05/2019, superseded version 2.0 which was used when recruitment started. The changes included lowering the minimum visual acuity threshold from Snellen 6/24, LogMAR 0.64 or 53 letters to Snellen 6/60, LogMAR 1.04 or 33 letters, the addition of interviews with healthcare professionals to address objective B and postal newsletters for participants.

## Discussion

Recruitment started on 31/07/2018. Recruitment and index test data collection has been challenging, primarily due to technical difficulties.

- During set-up, sites queried insurance, warranties and guarantees for liability of high-value equipment.
- Study staff were inexperienced in setting-up multiple device management software to maintain a large number of iPod devices.
- Distributing study equipment has been affected by restrictions on transporting devices containing lithium ion batteries by air.
- Mobile phone signal coverage at participant’s homes and at NHS sites is limited. The mVT and MBT applications require internet connectivity by the MiFi device. Difficulties with internet connectivity is a common reason for home eye test data not being transmitted.
- Faults and updates to software applications and Apple operating systems have caused interruptions to the availability of the index tests at participants’ homes.

Monitoring adherence to weekly home-monitoring is an important consideration. Following up participants who are not testing is time-consuming.

The results of this study will fill a gap in knowledge regarding the test-accuracy of home-monitoring for diagnosis of nAMD reactivation and the practicality of using home-monitoring with electronic devices in this patient group. After publication of the study results, the anonymised data will be made available upon reasonable request to the Sponsor institution, The Queen’s University of Belfast. The protocol includes a statement on data sharing [30].

### Summary

#### What was known before

- Wet age-related macular degeneration (nAMD) is the leading cause of vision loss in people aged ≥50 years. Whilst nAMD lesions can become inactive following treatment, relapse is common.
- Patients may have many months without requiring treatment and regular monitoring visits place a significant burden on NHS Hospital Eye Service (HES) out-patient clinics.
- Self-monitoring at home for patients with inactive disease would be more convenient and less costly for both patients and the NHS. HES clinic appointments could be given only to patients likely to require treatment or to newly referred patients.

#### What this study adds

- The MONARCH study is a multi-centre diagnostic test-accuracy cohort study evaluating three home-monitoring tests: two electronic tests using an iPod and one paper journal.
- The objectives of the MONARCH study are to estimate the test accuracy of the tests to self-monitor reactivation of nAMD, to determine the acceptability of the tests to patients and carers, to explore whether inequalities exist in recruitment, and to provide pilot data for using the same home-monitoring tests to detect conversion to nAMD in fellow eyes.

## Supplementary information

Supplemental Information

## References

[1] Colijn JM, Buitendijk GHS, Prokofyeva E, Alves D, Cachulo ML, Khawaja AP (2017). Prevalence of age-related macular degeneration in Europe: the past and the future. Ophthalmology..

[2] Writing Committee for the UKA-RMDEMRUG. (2014). The neovascular age-related macular degeneration database: multicenter study of 92 976 ranibizumab injections: report 1: visual acuity. Ophthalmology..

[3] Trevino R (2008). Recent progress in macular function self-assessment. Ophthalmic Physiol Opt.

[4] Chew EY, Clemons TE, Bressler SB, Elman MJ, Danis RP, Domalpally A (2014). Randomized trial of the ForeseeHome monitoring device for early detection of neovascular age-related macular degeneration. The HOme Monitoring of the Eye (HOME) study design - HOME Study report number 1. Contemp Clin trials.

[5] Kaiser PK, Wang YZ, He YG, Weisberger A, Wolf S, Smith CH (2013). Feasibility of a novel remote daily monitoring system for age-related macular degeneration using mobile handheld devices: results of a pilot study. Retin (Phila, Pa).

[6] Bittner AK, Torr-Brown S, Arnold E, Nwankwo A, Beaton P, Rampat R (2014). Improved adherence to vision self-monitoring with the vision and memory stimulating (VMS) journal for non-neovascular age-related macular degeneration during a randomized controlled trial. J Clin Exp Ophthalmol.

[7] Alster Y, Bressler NM, Bressler SB, Brimacombe JA, Crompton RM, Duh YJ (2005). Preferential Hyperacuity Perimeter (PreView PHP) for detecting choroidal neovascularization study. Ophthalmology..

[8] Wang YZ (2001). Effects of aging on shape discrimination. Optom Vis Sci.

[9] Wang YZ, He YG, Mitzel G, Zhang S, Bartlett M (2013). Handheld shape discrimination hyperacuity test on a mobile device for remote monitoring of visual function in maculopathy. Investig Ophthalmol Vis Sci.

[10] Wang YZ, Morale SE, Cousins R, Birch EE (2009). Course of development of global hyperacuity over lifespan. Optom Vis Sci.

[11] Wang YZ, Wilson E, Locke KG, Edwards AO (2002). Shape discrimination in age-related macular degeneration. Investig Ophthalmol Vis Sci.

[12] Wang Y-Z, He Y-G, Csaky KG, Mitzel G, Hernandez K, Zhang S (2015). Diabetic retinopathy and the MyVisionTrack® App (DRAMA) study. Investigative Ophthalmol Vis Sci.

[13] Winther C, Frisen L (2015). Self-testing of vision in age-related macula degeneration: a longitudinal pilot study using a smartphone-based rarebit test. J Ophthalmol.

[14] Winther C, Frisen L (2015). New rarebit vision test captures macular deficits hidden to acuity tests. Acta Ophthalmologica..

[15] Pitrelli Vazquez N, Harding SP, Heimann H, Czanner G, Knox PC (2018). Radial shape discrimination testing for new-onset neovascular age-related macular degeneration in at-risk eyes. PLoS One..

[16] Good Clinical Practice. https://www.nihr.ac.uk/health-and-care-professionals/learning-and-support/good-clinical-practice.htm.

[17] Whiting PF, Rutjes AW, Westwood ME, Mallett S, Deeks JJ, Reitsma JB (2011). QUADAS-2: a revised tool for the quality assessment of diagnostic accuracy studies. Ann Intern Med.

[18] Obuchowski NA, McClish DK (1997). Sample size determination for diagnostic accuracy studies involving binormal ROC curve indices. Stat Med.

[19] Macular Photocoagulation Study Group. Risk factors for choroidal neovascularization in the second eye of patients with juxtafoveal or subfoveal choroidal neovascularization secondary to age-related macular degeneration. Arch. Ophthalmol. (Chicago, Ill: 1960). 1997;115:741–7. 10.1001/archopht.1997.01100150743009 9194725

[20] Cachulo L, Silva R, Fonseca P, Pires I, Carvajal-Gonzalez S, Bernardes R (2011). Early markers of choroidal neovascularization in the fellow eye of patients with unilateral exudative age-related macular degeneration. Ophthalmologica J Int d’ophtalmologie Int J Ophthalmol Z fur Augenheilkd.

[21] Pieramici DJ, Bressler SB (1998). Age-related macular degeneration and risk factors for the development of choroidal neovascularization in the fellow eye. Curr Opin Ophthalmol.

[22] Solomon SD, Jefferys JL, Hawkins BS, Bressler NM (2007). Incident choroidal neovascularization in fellow eyes of patients with unilateral subfoveal choroidal neovascularization secondary to age-related macular degeneration: SST report No. 20 from the Submacular Surgery Trials Research Group. Arch Ophthalmol.

[23] Bossuyt PM, Reitsma JB, Bruns DE, Gatsonis CA, Glasziou PP, Irwig L (2015). STARD 2015: an updated list of essential items for reporting diagnostic accuracy studies. BMJ..

[24] Braun V, Clarke V. Successful qualitative research: Sage Publications; 2013. https://uk.sagepub.com/en-gb/eur/successful-qualitative-research/book233059.

[25] Pope C, Mays N. Qualitative research in health care. In: Pope C, Mays N. editors. Analysing Qual Data. BMJ. Oxford, BMJ Books, Blackwell Publishing; 2006.

[26] Luijkx K, Peek S, Wouters E (2015). “Grandma, You Should Do It—It’s Cool” older adults and the role of family members in their acceptance of technology. Int J Environ Res Public Health.

[27] May T, editors. Qualitative research in action. London: Sage Publications Ltd.; 2002.

[28] Yardley L. Dilemmas in qualitative health research. Psychol Health. 2000;15:215–28.

[29] Tong A, Sainsbury P, Craig J (2007). Consolidated criteria for reporting qualitative research (COREQ): a 32-item checklist for interviews and focus groups. Int J Qual Health Care.

[30] Monitoring for neovascular AMD Reactivation at Home: the MONARCH study. 2017. https://www.journalslibrary.nihr.ac.uk/programmes/hta/159702/#/.

