# Supplementary material for: Monitoring for neovascular age-related macular degeneration (AMD) reactivation at home: the MONARCH study
Source: Eye (Lond). 2020 May 4;35(2):592–600. doi: 10.1038/s41433-020-0910-4 (PMC8027627; doi:10.1038/s41433-020-0910-4)
Supplement: Supplementary file 1 — Supplemental Information [file 41433_2020_910_MOESM1_ESM.docx]

**Supplementary information 1**

**Retinal image collection**

Participants are asked to consent (optional) for their retinal images to be collected. With consent, all retinal images taken as part of usual care are uploaded in a linked-anonymised form to The Central Angiographic Research Facility, Belfast (CARF) for storage and use in future ethically approved research.

**Patient approach**

Patients with nAMD are identified and approached according to local site procedures. All potential participants receive an invitation letter and participant information leaflet describing the study and have time to consider whether to participate. A member of the local research team answers any questions the patient has. Patients who are ‘willing in principle’ to take part are provide verbal consent to attend a ‘further information and training session’ at the hospital led by a member of the local research team. At this session, the patient is shown the equipment and how to do the tests. The local research team confirms eligibility and takes written informed consent if the patient decides to participate.

**Qualitative subgroups**

1. Participants carrying out home-monitoring tests (n=~25 x 3 sites: ~75) including observations of the physical and social context of the home environment and the presence and interaction of a carer. Approximately five of the 25 participant interviews at each site will be with participants who state that they do not have a carer and illuminate further the role of carers in home-monitoring.

2. Family carers or friend carers of participant interviewees are also interviewed separately (n=~60 carer interviews).

3. Test data will be used to create a sampling frame of participants who complete one, two or three tests, who stop-and-start and who drop out. Up to ~20 participants and ~20 carers (n=~40 interviewees in total) from these subgroups will be invited to participate.

4. Participants (and their carers) who decline to take part in home-monitoring testing are being interviewed (~5 patients and 5 carers per site x 3: n=~15 patients; n=~15 carers).

**Methods to minimise bias.**

*Bias due to selection of participants*

Bias is avoided by using a cohort study design and recruiting a representative sample of eligible patients. Staff may not recruit consecutive eligible patients but factors such as absence on leave or other logistical issues will not be associated with the characteristics of patients. When staff are available, research teams are expected to invite consecutive eligible patients to take part and hence recruit a representative sample of patients. The exclusion criteria are appropriate, i.e. they would prevent a person self-monitoring using one or more of the tests even if the test(s) were implemented by the NHS (if shown to detect nAMD reactivation accurately).

*Bias in the assessment of the index tests*

Bias is avoided because index tests will be ‘scored’ without knowledge of the results of the reference standard; we will compare index test results for the weeks before a monitoring visit with the reference standard classification at the visit. For analyses of test performance, we will pre-specify the methods for classifying lesion status from index test results based on the knowledge of the distribution of scores and expert judgements about weights for false positive and false negative misclassifications. We cannot specify test thresholds at the outset because there are no available data to inform these definitions.

*Bias in the assessment of the reference standard*

Bias is avoided by ensuring that the reference standard is assessed without knowledge of the results of the index tests, which are transmitted by the internet or post directly to the coordinating centre. Participants may have impression of whether their vision has deteriorated from carrying out the index tests at home, and they may communicate this impression to their ophthalmologists. However, neither the participants nor the ophthalmologists will have any quantitative information about test performance. The reference standard represents a usual care decision about the reactivation of nAMD and, although this decision will not always be accurate [1], it can reasonably be considered likely to classify participants correctly with respect to reactivation of nAMD.

*Bias due to exclusion of participants or inappropriate intervals between the times of index testing and the reference standard*

Bias is avoided by ensuring the analysis includes all follow-up visits for which the reference standard is assessed and by describing the time intervals between index tests and the reference standard. We will also account for all patients recruited into the study e.g. using a flow diagram and tables as appropriate. We will acknowledge potential differences between participating centres and present information that may characterise this, e.g. variation in methods used to obtain the reference standard and the centre-specific rate of reactivation of nAMD.

**Study management and monitoring**

Preparation of study documents, site initiation and training, day-to-day running of the study and monitoring of sites and participant adherence according to the central monitoring plan is managed by CTEU Bristol. The qualitative component of the study is managed by CT and MD with input from REH at Queen’s University of Belfast. Study equipment is set-up and maintained by CT at Queen’s University of Belfast, with input from REH and CTEU Bristol. A PPI group to evaluate study materials and provide feedback on the participant burden of the study is coordinated by CT and REH in Belfast. A study management group (SMG) meets regularly to oversee progress. A study steering committee (SSC) oversees the overall study, receives reports and recommendations from the SMG and has ultimate responsibility for periodic recommendations about continuing the study.

**Plan for statistical analysis (Objective A)**

We propose preliminary exploratory analyses to identify how best to maximise performance (both for index tests and combined home-monitoring data), inspecting receiver operator characteristic (ROC) curves that characterise the performance of different ways of aggregating scores from multiple tests occasions for an index test. In order to minimise bias, we will pre-specify the method of choosing test thresholds.

The number of home-monitoring assessments between each clinic visit will vary by visit and by participant due the timing of the hospital visits and how closely the participant adheres to the weekly monitoring schedule. We will create a summary measure for the home-monitoring scores obtained between two hospital visits and will use weighting in the analysis to reflect the precision of the summary (i.e. based on the number of scores contributing to the summary). The choice of the appropriate summary measure will be decided in discussion with the clinicians on the team and by examining the profiles of participants’ scores masked to other information or test results.

SPIRIT diagram of study timepoints and data collection schedule; objectives A, C and D.

|  | **Enrolment** | **Consent** | **Post-consent** | | |
| --- | --- | --- | --- | --- | --- |
| **TIMEPOINT** | ***-t_1_*** | **0** | ***Throughout follow-up*** | | |
| **LOCATION** |  |  | ***Home (weekly)*** | ***Before HES visit*** | ***During HES visit*** |
| **ENROLMENT:** |  |  |  |  |  |
| **Eligibility**  **pre-screen** | X |  |  |  |  |
| **Information and training session** | X |  |  |  |  |
| **Eligibility check** | X |  |  |  |  |
| **Consent** |  | X |  |  |  |
| **Study equipment provided** |  | X |  |  |  |
| **Most recent visual acuity** |  | X |  |  | X |
| **Smoking status** |  | X |  |  |  |
| **Medical history** |  | X |  |  |  |
| **Ocular history** |  | X |  |  | X |
| **Retinal imaging**** |  | X |  |  | X |
| **TESTS:** |  |  |  |  |  |
| **KeepSight Journal** |  |  | X |  |  |
| **My Vision Track** |  |  | X |  |  |
| **Multibit Test** |  |  | X |  |  |
| **Patient-reported (PR) visual function** |  |  |  | X |  |
| **PR experience of home eye testing** |  |  |  | X |  |
| **PR problems with home eye testing** |  |  |  | X |  |
| **PR problems with test equipment** |  |  |  | X |  |
| **AMD treatment since last visit** |  |  |  |  | X |
| **Management decision*** |  |  |  |  | X |

AMD = age-related macular degeneration; HES = hospital eye service; PR = patient-reported

*Reference standard performed by ophthalmologist at routine follow-up visits where a management decision is made (AMD active, inactive or uncertain). There are no mandated assessments required to determine the reference standard in this study.

**Details of retinal images taken ≤ 4 weeks of consent date. Retinal images sent to Central Angiographic Research Facility only for participants who consent to this aspect of the study.

**References**

1. Reeves BC, Scott LJ, Taylor J, Harding SP, Peto T, Muldrew A, et al. Effectiveness of Community versus Hospital Eye Service follow-up for patients with neovascular age-related macular degeneration with quiescent disease (ECHoES): a virtual non-inferiority trial. BMJ Open. 2016;6(7). doi: 10.1136/bmjopen-2015-010685.
